# Supplementary material for: Patterns of Intron Gain and Loss in Fungi
Source: PLoS Biol. 2004 Nov 30;2(12):e422. doi: 10.1371/journal.pbio.0020422 (PMC532390; doi:10.1371/journal.pbio.0020422)
Supplement: Table S1 — Also available at http://genes.mit.edu/NielsenEtAl/. (4.3 MB ZIP). [file pbio.0020422.st001.zip › NielsenEtAl/html/229.html]

AN7444.1.NCU04241.1.MG06874.1.FG10274.1


```
 CLUSTAL W (1.82) Multiple Sequence Alignments - Introns Inserted


Sequence 1: NCU04241.1	600 aa
Sequence 2: FG10274.1	564 aa
Sequence 3: MG06874.1	679 aa
Sequence 4: AN7444.1	523 aa
Alignment Length: 712 aa
Number Identitical Residues: 261 aa
Alignment Score (without introns) 13129


MG06874.1 	MFSSGSGSG~KKVPPQ0Q~EMVQVD1RSD2S~AS---~--------PFWRQPSTSARS~R
NCU04241.1	MFSSSSSSG~KKASP-~Q0AMMSLR~QQE~M~VQVGI2SGSPFWRAPQKDAPLKKTTT~R
FG10274.1 	MWSSSGS-G~KKSAQ-~S0PHCRRR~WSK~L0IG---~------------------QS1H
AN7444.1  	MATRKVQQQ0TRQSS-~S~PFWKAR~SVS~Q~NY---~--------------ERTPNS~P
          	* :    .  .: .   .          .                         .. :  

MG06874.1 	DGSKVVGAAYSHADMLKIDAL2N~VSGSSSRPQSRLHTPPSAPLSKPPKNIMTSPPSRPA
NCU04241.1	DSGKVVGAAYSHADMLKFDSL2N~LSNAGSRPRT---PPPSS--SALGRTTSNSTASPHI
FG10274.1 	GSGLIVGAAYSHADMLKFDSL2N~SSNS-SRPRT---PPSSS--STKIRIQEASPPVKAT
AN7444.1  	DQSLIVGASYSHANIP-----~N2GLSQHTRPRT---PPRSS-----PNNSSHASPSTRR
          	. . :***:****::       *   .  :**::   .* *:      .    :..    

MG06874.1 	PG-LGSPPIKSYVNFLSRTNDDWKMDEGDDYEDYGDDD-----------GDDFGLPSLSS
NCU04241.1	KG-VTSPPFQNYMNFITSTNEDWAADDADDVYAYEDDG-----------DDDFGLPSLSS
FG10274.1 	PGKRGSPPYKSYINFLSNTNDDWKADE-DEMMGYEDDD-----------GDDFGLPSLSN
AN7444.1  	PGIWVASPRASYSGIMNQELGPWDHGNPAGKIGDDDDDDVGEDIVFDDDEDEFGLPSIAS
          	 *   :.*  .* .::.     *  .:        **.. ...   ... *:*****::.

MG06874.1 	TKRRNTRRMAEEQ----ERSVSHSG---VTSP---LAMRQFEDTGDIAIERPSSAYPISK
NCU04241.1	MKRR-SRRIAAQT----EQGTSRGT---ATNTLTDATGRRYSDSADIAIERPASTYPMPK
FG10274.1 	TKRR-TRRIATQNRA--ESSTLSPS---TEGPSPTFLTRRYSNSADIAIERPAPTYPMPK
AN7444.1  	MRKRQGRQADVSRFKPVETPTFKSDSRPSTLDVNLPNNRQRANSSDIAEERDIPLYPTAR
          	 ::*. *:   .   . *  .    : .          *:  ::.*** **  . ** .:

MG06874.1 	KSEGKILRPQYKDILKD~PANALHLISHPSTPH~DATPKEIDAANSRITRINKFKKILQS
NCU04241.1	KTEGKILRPQYRDILRD~PANALHLINHPSVPP~NASQKEIDAVNSRITRINKFKKILQA
FG10274.1 	KSEGKILRPQYKEILK-~--------------A1NATPKETDAINSRITRINKFKKLLQA
AN7444.1  	KSDGKILRPQYKDILKD1PANALNLINHVAPPK~GASSKDMDAYTSRISRINKFKRLLQA
          	*::********::**:. .:.:    .  : .  .*: *: ** .***:******::**:

MG06874.1 	SSIPLTELRALAWSGVPEEVRAMTWQLLLSYLPTSSERRVAVLERKRKEYLDGVRQAFES
NCU04241.1	STIPLNDLRALAWSGVPEEVRAMTWQLLLSYLPTSSERRVATLERKRKEYLDGVRQAFER
FG10274.1 	STISLPDLRQLAWSGVPQEVRAITWQLLLSYLPANSERRVATLERKRKEYLDGVRQAFER
AN7444.1  	STVPLQELRNLAWSGVPDEVRAMTWQLLLGYLPTNSERRIATLERKRKEYLDGVRQAFER
          	*::.* :** *******:****:******.***:.****:*.***************** 

MG06874.1 	GSGVKPSNSSSNAASTPGRGGRGLDEAIWHQISIDVPRTNPHIELYGYEATQRSLERILY
NCU04241.1	AGGAPPP--STGKGGGG---NRGLDEAIWHQISIDVPRTNPHIELYGYEATQRSLERILY
FG10274.1 	GGGPTTT--SANTASTGR--TRGLDEAIWHQISIDVPRTNPHIELYSYEATQRSLERILY
AN7444.1  	SNAATTRE-NSRSTDTGR--GRGLDEAIWHQISIDVPRTSPHIQLYSYEATQRSLERILY
          	...  . . .:   .      ******************.***:**.*************

MG06874.1 	VWAVRHPASGYVQGINDLVTPFWQVFLGTYITDPDIERGMDPGQLPRAVLDAVEADTFWC
NCU04241.1	VWAVRHPASGYVQGINDLVTPFWQVFLGTYITDPDIESGMDPGQLPRAVLDAVEADTFWC
FG10274.1 	LWAVRHPASGYVQGINDLVTPFWQVFLGIYIGDPDIESGMDPGQLPKSVLDAVEADSFWC
AN7444.1  	VWAIRHPASGYVQGINDLVTPLFQVFLGVYVTDLNVEEGMDPGQLPRSVLDAVEADSFWC
          	:**:*****************::***** *: * ::* ********::********:***

MG06874.1 	LTKLLDGIQDHYIVAQPGIQRQVSALRDLTARIDEQLAKHLERENVEFIQFSFRWMNCLL
NCU04241.1	LTKLLDGIQDHYIVAQPGIQRQVAALRDLTQRIDAGLAKHLEEENVEFIQFSFRWMNCLL
FG10274.1 	LTKLLDGIQDHYIVAQPGIQRQVTALRDLTARIDANLSKHLEQEGIEFIQFSFRWMNCLL
AN7444.1  	LTKLLDGIQDNYIYAQPGIHRQVRALRDLTMRIDATLAKHLEQEGVEFMQFSFRWMNCLL
          	**********:** *****:*** ****** ***  *:****.*.:**:***********

MG06874.1 	MREISVKNTIRMWDTYM0AEENGFSEFHL~YVCAAFLVKWSAKLTKMDFQEIMMFLQSLP
NCU04241.1	MREISVKNTIRMWDTYM0AEEQGFSEFHL~YVCAAFLVKWSDKLVKMDFQEIMMFLQSLP
FG10274.1 	MREFSVKNTIRMWDTYL0AEEQGFSEFHL~YVCAAFLVKWSDKLLDMDFQEIMMFLQSLP
AN7444.1  	MREMSVRNTIRMWDTYM0AEEQGFSRFHL2K-----------------------------
          	***:**:*********: ***:***.***                               

MG06874.1 	TKEWTEKDIELLLSEAFIWQSLYKGSSAHLRAPSSSRSPSMGFQL2TKVDADVQMDSCRG
NCU04241.1	TREWTEKDIELLLSEAYIWQSLFKGSSAHLKGQPSSRANSLNFQL~--------------
FG10274.1 	TKGWTEKDIELLLSEAFIWQSLFKGSAAHLKGQP-AREPVTNLQL~--------------
AN7444.1  	---------------------------------------------~--------------
          	                                                            

MG06874.1 	HKVLSHVYVSRHNMLVVEATARRDISETRICYGACQYYRKDAGLHYDMHGEPVYDDRMLD
NCU04241.1	------------------------------------------------------------
FG10274.1 	------------------------------------------------------------
AN7444.1  	------------------------------------------------------------
          	                                                            

MG06874.1 	DVVFMNV
NCU04241.1	-------
FG10274.1 	-------
AN7444.1  	-------
          	
```
